# Double left brachiocephalic vein in an adult patient who underwent cardiac surgery: a case report

**DOI:** 10.1186/s13019-021-01630-8

**Published:** 2021-08-28

**Authors:** Kimihiro Kobayashi, Tetsuro Uchida, Yoshinori Kuroda, Atushi Yamashita, Eiichi Ohba, Shingo Nakai, Tomonori Ochiai, Mitsuaki Sadahiro

**Affiliations:** Second Department of Surgery, Faculty of Medicine, Yamagata University, 2-2-2 Iida-Nishi, Yamagata, 990-9585 Japan

**Keywords:** Vascular anomaly, Left brachiocephalic vein, Cardiac surgery, Case report

## Abstract

**Background:**

A double left brachiocephalic vein is an extremely rare venous anomaly.

**Case presentation:**

Herein, we present the case of a 79-year-old woman with a double left brachiocephalic vein who underwent cardiac surgical procedures. The normal left brachiocephalic vein was patent, and the accessory left brachiocephalic vein passed across the heart and aorta in front of the pericardium and drained into the superior vena cava. She underwent surgical ligation of the accessory left brachiocephalic vein, followed by an aortic valve replacement and coronary artery bypass grafting. Her postoperative recovery was uneventful, without any venous complications from the ligation of the accessory vein. The patient is doing well one year after the surgery.

**Conclusions:**

The presence of double left brachiocephalic veins should be recognized before cardiac surgery in order for us to avoid intraoperative technical issues concerning this venous anomaly and unpredictable intraoperative bleeding due to injury of the accessory left brachiocephalic vein.

## Background

A double left brachiocephalic vein (BCV) is a rare vascular anomaly, and only a few cases have been reported [1]. In addition to the normal left BCV, the accessory left BCV passes various courses, such as the retroaortic, preaortic, retrotracheal, and retroesophageal, and drains into the superior vena cava [1, 2]. Herein, we present the case of a patient with an accessory left BCV passing a preaortic course, who underwent cardiac surgical procedures.

## Case presentation

**Fig. 1:**
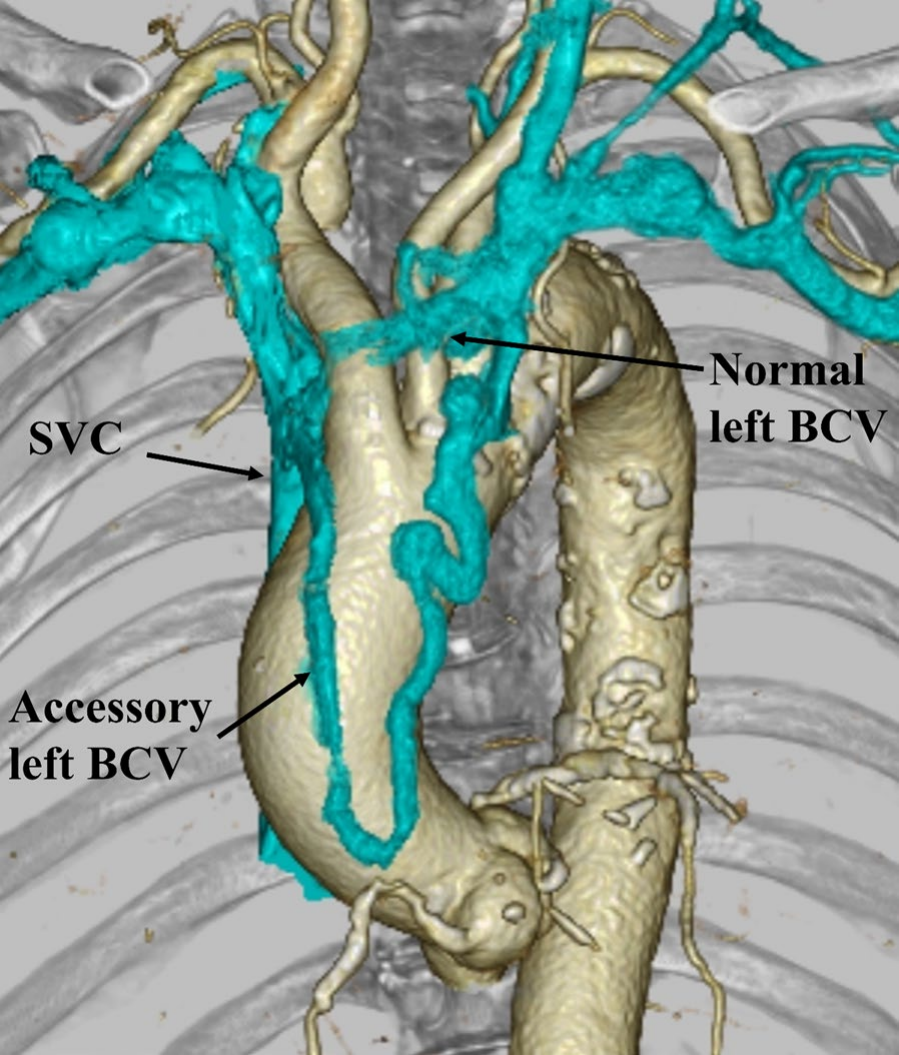
The 3D-reconstructed image of the double left brachiocephalic vein. BCV, brachiocephalic vein; SVC, superior vena cava

**Fig. 2:**
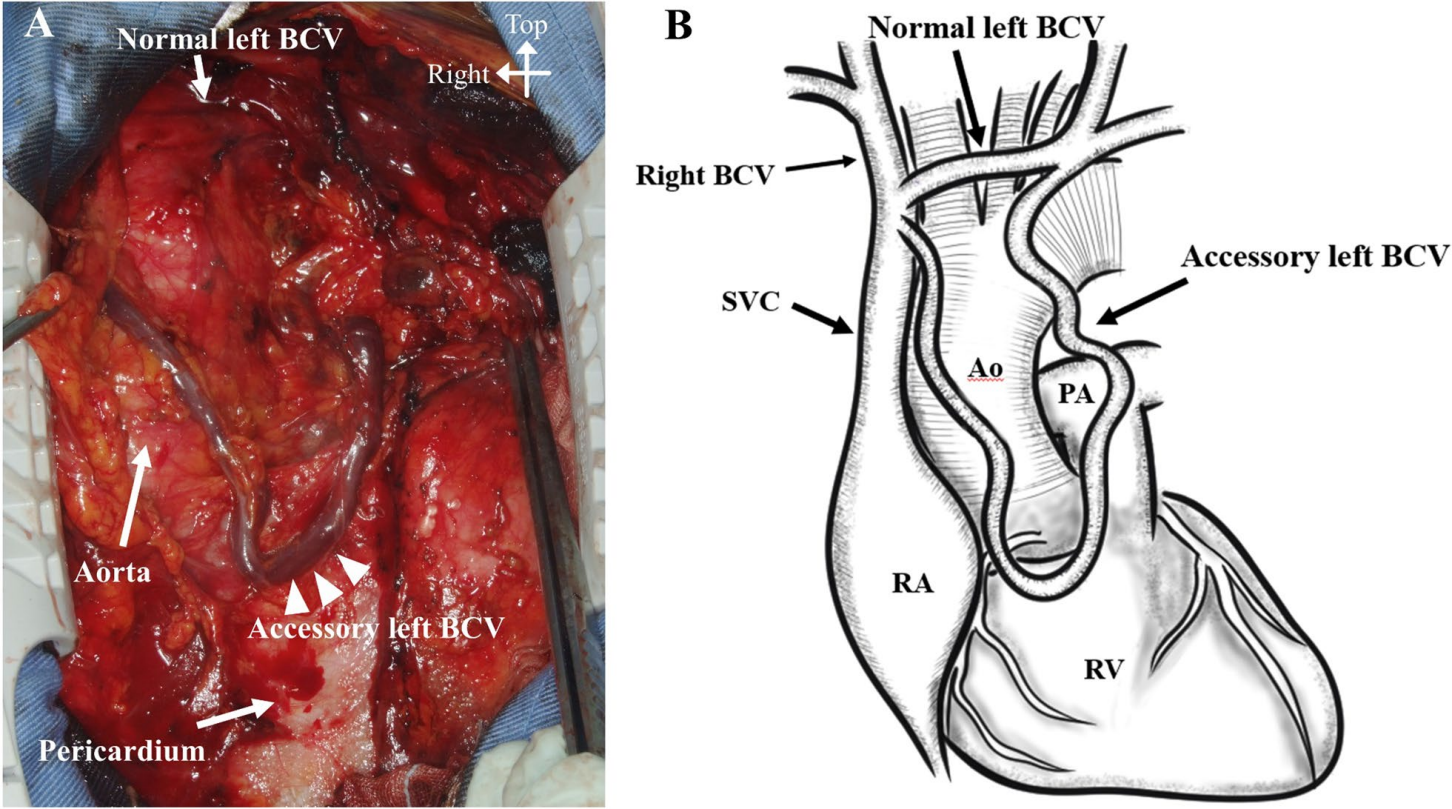
**A** The intraoperative image of the accessory left brachiocephalic vein passing across the ascending aorta and heart in front of the pericardium. **B** Schematic drawing of the double left brachiocephalic vein with a preaortic course. Ao, ascending aorta; BCV, brachiocephalic vein; PA, pulmonary artery; RA, right atrium; RV, right ventricle; SVC, superior vena cava

**Fig. 3:**
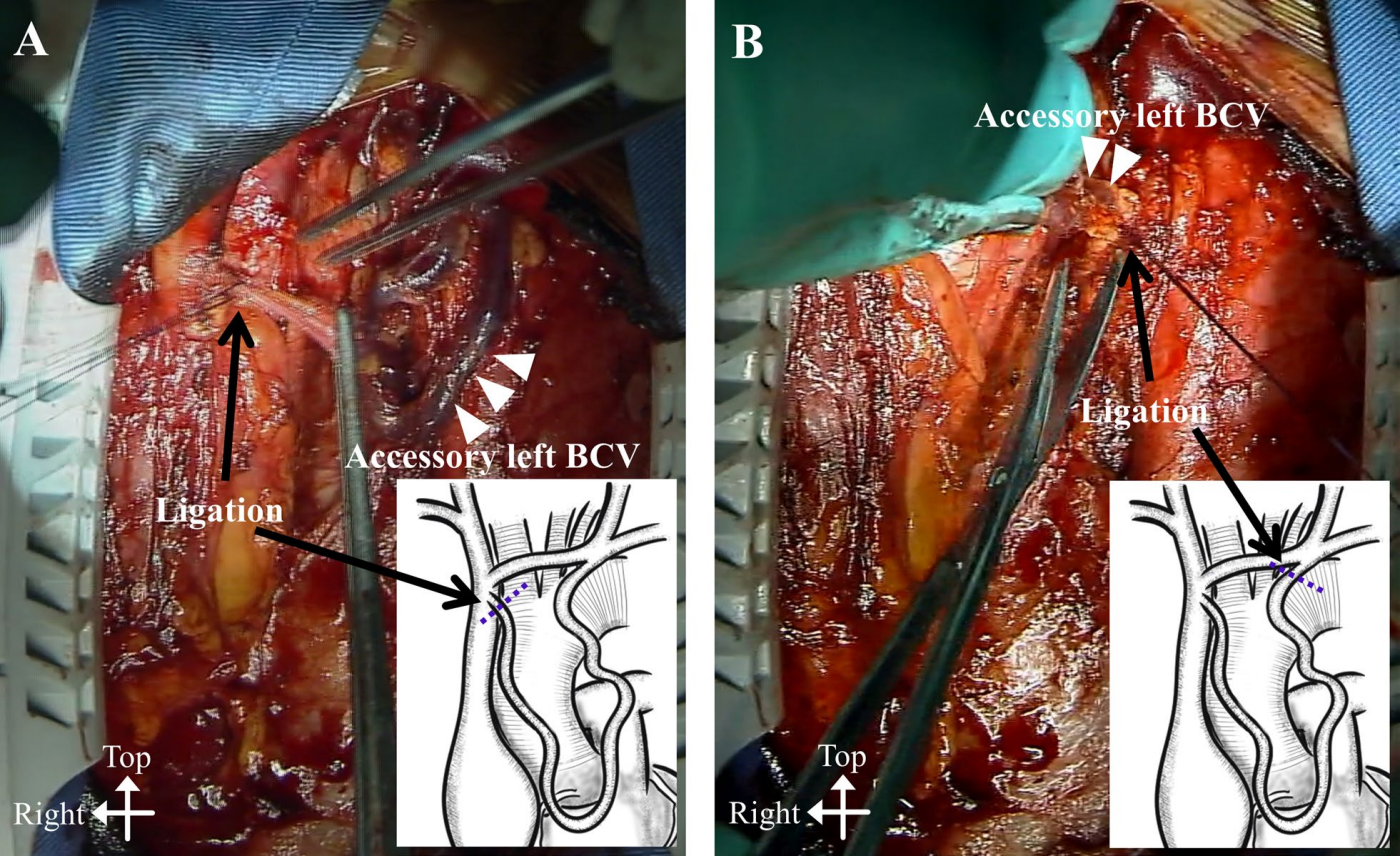
Intraoperative images showing the ligation of the accessory left brachiocephalic vein close to its junction with the **A** superior vena cava and **B** normal left brachiocephalic vein. BCV, brachiocephalic vein

A 79-year-old woman with severe aortic valve stenosis and coronary artery disease was scheduled for cardiac surgery. Preoperative contrast-enhanced computed tomography (CT) incidentally revealed a double left BCV (Fig. 1). The left subclavian vein and left internal jugular vein merged to form the left BCV, which then divided into two branches: the normal and accessory left BCVs. The accessory left BCV was tortuous and passed across the front of the ascending aorta. Both the left BCVs drained into the superior vena cava at a level higher than the azygos vein. Following a median sternotomy, anterior mediastinal tissue was carefully dissected and the accessory left BCV was easily identified. The left accessory BCV passed across the heart and ascending aorta in front of the pericardium and drained into the superior vena cava at the same level as the normal left BCV (Fig. 2). Prior to opening the pericardium, the accessory left BCV was surgically ligated and divided (Fig. 3), because the normal left BCV was patent. Subsequently, an aortic valve replacement and coronary artery bypass grafting to the left anterior descending artery using the left internal mammary artery was performed. The postoperative course was uneventful, without any complications from the ligation of the accessory left BCV, such as upper body congestion. Histopathological examination of the resected wall of the accessory left BCV showed normal structures.

## Discussion and conclusions

A double left BCV was first described by Subirana in 1986 [3]. Since then, there have been few additional reports of this venous anomaly. Due to its rarity, the incidence and developmental mechanisms are not well understood [1]. The normal left BCV originates in the transverse channel formed between the precardinal veins during the 4th to 8th week of fetal development [1, 2, 4]. Some authors have speculated that double transverse channels and their remnants might lead to the formation of a double left BCV [1, 2].

Clinically, most double left BCVs, by themselves, do not affect the patient’s condition. However, when performing the insertion of central venous catheters and electrical leads through the left subclavian vein, there may be some technical difficulties and a potential risk of venous injury due to an undiagnosed accessory left BCV [1, 2, 5].

In our present case, the accessory left BCV was diagnosed preoperatively. Therefore, we recognized the abnormal preaortic pathway of this accessory left BCV and patency of the normal left BCV. This enabled us to safely ligate the accessory left BCV prior to the intended cardiac surgical procedures. However, if the normal left BCV is hypoplastic, severely stenotic [5], or occluded [6], preservation of the accessory left BCV throughout the procedure, or transection followed by reconstruction, should be mandatory to avoid upper body congestion. In patients with an undiagnosed double left BCV, there is the risk of unexpected intraoperative bleeding due to injury of the accessory left BCV, particularly if it has a preaortic course.

For the establishment of cardiopulmonary bypass, there are some technical issues in patients with double left BCVs. There is a potential risk of inadequate venous drainage due to the obstruction of the aberrant BCV opening at the superior vena cava by the venous cannula itself [4]. Differentiating between the accessory left BCV and persistent left superior vena cava is also important to determine the necessity of additional venous cannulas for use in a cardiopulmonary bypass.

Preoperative venous evaluation is important in patients undergoing cardiac surgical procedures [1, 4]. Contrast-enhanced multidetector CT images are useful for the precise, preoperative diagnosis of left BCV anomalies [4]. The possibility of misidentifying an accessory left BCV as a mediastinal lymph node on unenhanced CT has been suggested as a potential risk [4, 7].

Although the double left BCV is rare, cardiovascular surgeons should be aware of this venous anomaly to avoid several intraoperative complications.

## Data Availability

The data are not available for public access due to patient privacy concerns but are available from the corresponding author upon reasonable request.

## Abbreviations

BCV: Brachiocephalic vein
CT: Computed tomography
